# Organisational and advance care planning program characteristics associated with advance care directive completion: a prospective multicentre cross-sectional audit among health and residential aged care services caring for older Australians

**DOI:** 10.1186/s12913-021-06523-z

**Published:** 2021-07-16

**Authors:** Karen M. Detering, Craig Sinclair, Kimberly Buck, Marcus Sellars, Ben P. White, Helana Kelly, Linda Nolte

**Affiliations:** 1 Advance Care Planning Australia, Austin Health, Melbourne, Australia; 2 Faculty of Health, Arts and Innovation, Swinburne University of Technology, Hawthorn, Australia; 3 Centre of Excellence in Population Ageing Research, University of New South Wales, Sydney, Australia; 4 School of Psychology, University of New South Wales, Sydney, Australia; 5 Department of Health Services Research & Policy, Research School of Population Health, College of Health & Medicine, The Australian National University, Canberra, Australia; 6 Australian Centre for Health Research Law, Faculty of Law, Queensland University of Technology, Brisbane, Australia; 7 Peter MacCallum Cancer Centre, Melbourne, Australia

**Keywords:** Advance care planning, Advance care directives, Prevalence, Audit, General practice, Hospital, Aged care

## Abstract

**Background:**

Advance care planning (ACP) and advance care directive (ACD) completion improve outcomes for patients, family, clinicians and the healthcare system. However, uptake remains low. Despite increasing literature regarding organisational-level ACP characteristics leading to success, there is a lack of data measuring the impact of these factors on ACD prevalence.

**Methods:**

A prospective multi-centre, cross-sectional audit of health records among older Australians accessing general practices (GP), hospitals and residential aged care facilities (RACF) was undertaken to describe organisational and ACP-program characteristics across services, document ACD prevalence, and assess organisation-level predictors of ACD prevalence. Organisational-level data included general and ACP-program characteristics. Patient/resident data included demographics and presence of ACDs.

**Results:**

One hundred organisations (GP = 15, hospitals = 27, RACFs = 58) participated, contributing data from 4187 patient/resident health records. Median prevalence of ACDs across organisations was 19.4%, (range = 0–100%). In adjusted models, organisational sector type was the strongest predictor of ACD prevalence, with higher rates in RACFs (unadjusted 28.7%, adjusted 20.6%) than hospitals (unadjusted 6.4%, adjusted 5.8%) or GPs (unadjusted 2.5%, adjusted 6.6%). RACFs in regional and rural/remote areas had higher prevalence than metropolitan organisations. Organisations supported by government funding and those that were Not For Profit had higher prevalence than those that were privately funded, and organisations with an ACP program that had been implemented at least 3 years before data collection had higher prevalence than those with either no program or a more recent program.

**Conclusions:**

The median ACD prevalence was low, with substantial variation across organisations. Sector type was the strongest predictor, being highest in RACFs. Low prevalence rates, overall and in particular sectors, have implications for improvements. Further research into organisational factors associated with ACP/ACD completion is required.

## Introduction

An ageing population and the associated increase in chronic illness burden, especially in the later years and towards the end of life, poses system-wide healthcare challenges in Australia and internationally [1, 2]. Simultaneously, there is an increasing emphasis on empowering health and aged care service consumers to have greater control over treatment decisions, both now and in the future, with a shift to seeing comprehensive care as care that is consistent with individuals’ values, goals and informed preferences [3]. The importance of advance care planning (ACP) is increasingly being recognised as a marker of quality care and has become a key priority for health and aged care. In Australia, legislation, policy and accreditation quality standards support the implementation of ACP across the health and aged care sectors [4–9].

ACP is a voluntary and iterative process of reflection and discussion that aims to clarify and share the person’s values and preferences, so these can guide medical treatment decision-making should the person subsequently lose decision-making capacity [10, 11]. ACP may also involve the legal appointment of a substitute decision-maker (SDM). The goal of ACP is to provide care consistent with the person’s known preferences [11–13]. Evidence has shown that ACP has important beneficial outcomes for patients, their families, healthcare staff and the healthcare system. These include improved quality of end-of-life care for patients, enhanced psychological outcomes and lessening of decision-making burden for bereaved family members, a reduction in moral distress for staff and better usage of resources with a potential reduction in costs for organisations and the broader health and aged care systems [13–18].

Whilst conversations about treatment preferences are essential, documentation of the outcomes of ACP discussions increases the likelihood that care provided will be consistent with the person’s preferences [14, 15]. Documentation also supports SDMs and clinicians when making treatment decisions on behalf of a person lacking decision-making capacity [7, 9, 11, 19]. However, for this to occur, documentation needs to be accessible at the point-of-care [20, 21], and utilised to develop medical treatment plans, where the person lacks decision-making capacity to participate in decisions about their treatment. The nature and scope of ACP documentation varies within Australia and internationally [7, 9, 13, 22]. In Australia, documentation includes advance care directives (ACD), a term encompassing documents recognised by jurisdiction-based legislation (statutory ACD: preferences for care or appointment of SDM) or common law (non-statutory ACDs) that are completed and signed by a competent adult [7, 9].

Despite the evidence, legislation, policy and quality standards supporting ACP and ACD completion, uptake remains low. A 2017 Australian ACD prevalence study of older people showed that only 30% of people had an ACD in their records at the point of care [23]. Whilst a similar prevalence rate has been reported in the USA [19], other countries generally report lower rates [24–26]. Yet literature also reports people often want to undertake ACP [13, 19, 24, 27, 28]. Further research is required to understand factors influencing this variation between actual documentation and the person’s wish to do so.

Implementation and evaluation of ACP interventions across multisector healthcare systems is required if full potential of ACP is to be achieved. The optimal methods for achieving widespread implementation of ACP across large populations and throughout complex multisector healthcare systems are poorly understood [29–31]. There are reports of successful ACP within individual services [18], across regions [32, 33] and within a single sector [34].There are also multiple published reviews examining individual, organisational and/or system-wide facilitators and barriers to ACP implementation, and these have looked at patient, family and provider views across various settings such as in aged care, primary care, in hospitals and within the community, and have included a range of people from healthy older people to patients with a range of serious illness [13, 35–40].

Key elements thought to be associated with successful ACP within organisations, across various settings include the provision of ACP training for staff, access to standardised consumer information and standardised ACD templates, clear delineation of staff roles and responsibilities, ideally outlined in organisational policy, adequate resourcing to support ACP and systems available for storage and retrieval of ACDs across multiple settings [13, 35, 36, 41, 42]. Despite the growing body of evidence as to what is needed to successfully implement ACP, there is a gap in the evidence relating to the measurement of important outcomes of ACP, such as ACD prevalence, at the service organisational-level, and characteristics that are associated with higher prevalence. Organisational support is key to successful implementation [43].

The aims of this study were to 1. describe the organisational and ACP-program characteristics across general practices, hospitals and residential aged care facilities (RACFs); 2. document the prevalence of ACDs within these organisations; and 3. to determine organisational-level and ACP-program predictors of ACD prevalence.

## Methods

### Study design

Data reported originate from the *National ACD prevalence study: a prospective multi-centre cross-sectional audit of health records among older Australians accessing health and residential aged care services*. As the full research protocol has been published elsewhere [44], a summary of the methodology is provided. This paper reports on the organisational and ACP program characteristics associated with ACD prevalence rates; person-level factors associated with ACD prevalence will be reported elsewhere.

Ethics approval was obtained from Austin Health Human Research Ethics Committee, Melbourne, Australia (ref: HREC/18/Austin/109) and organisation-specific approval was obtained where required. All methods were performed in accordance with the relevant guidelines and regulations.

### Participant organisations and recruitment

Recruitment occurred at the organisation level. Eligible organisations were accredited Australian general practices, public and private hospitals and RACFs, who were recruited from all eight Australian jurisdictions, through an online expression of interest process. Additional organisations were approached by the project team to promote sample representativeness across sectors and jurisdictions. All organisations that met eligibility criteria were included.

Organisations were expected to provide staff to audit 30 to 50 records of patients/residents aged 65 years and older attending their service. However, organisations with limited resourcing could request access to trained auditors provided by the project team, thereby enabling these organisations to participate. All data collectors were specifically trained in the audit methodology.

Data obtained from the patient/resident record audit included demographic data and the presence of one or more ACDs. Each audit took 20 to 30 min to complete. In hospitals and RACFs, health records for auditing were randomised from a list of all eligible people, whereas consecutive records were audited in general practices.

### Data collection

Organisation-level data was collected during the recruitment process via applicant self-report. Data included general characteristics (sector, jurisdiction, location, funding source), service size (number of beds, number of staff), presence of an ACP program (existence of an ACP program, and when implemented), and ACP program characteristics (availability of staff training in ACP, presence of ACP policy, written ACP resources available for patients or residents, the existence of mechanisms for ACD storage and accessibility, ACD templates available for use, and availability of specific funding for ACP).

Health record audit data were entered and stored on a secure cloud-based database specifically built for this project. Health records (paper and/or electronic records including the Australian “My Health Record” [45]) were searched for a maximum of 15 minutes for ACDs. A time of 15 min was selected in recognition that for documents to be useful in emergencies, they need to be easily located within a person’s record. For this study [44],and in line with Australian law [9]. ACDs were defined as formal documents recognised by either statutory legislation (1. statutory ACD: preferences for care; or 2. statutory ACD: substitute decision-maker (SDM) appointments) or common law (non-statutory ACD). People could have one or more of these documents present in their records.

### Outcome measures

The primary outcome measure was the mean ACD prevalence for each participating organisation. This value was determined by counting the number of patients/residents who had at least one ACD located within 15 minutes of the data collector accessing the health record. The ACD prevalence for each organisation was then calculated by dividing this number by the total number of records audited at that organisation.

### Statistical analysis

Variable recoding and statistical analyses were conducted using R Studio (version 1.3.1093). Organisation characteristics and ACP program data were summarised using frequencies and percentages. Due to substantial correlations between the individual ACP program measures and different patterns of implementation across organisational sector type, a summed measure of ACP program implementation was generated by adding up the number of program characteristics present within each organisation, (Maximum equals six – being training for staff, an ACP/ACD policy, written resources for clients, mechanisms to record presence of ACD, ACD template, and funding available for ACP activities) and categorised as ‘High’ (5 or more) and ‘Some or less’ (less than 5).

**Fig. 1:**
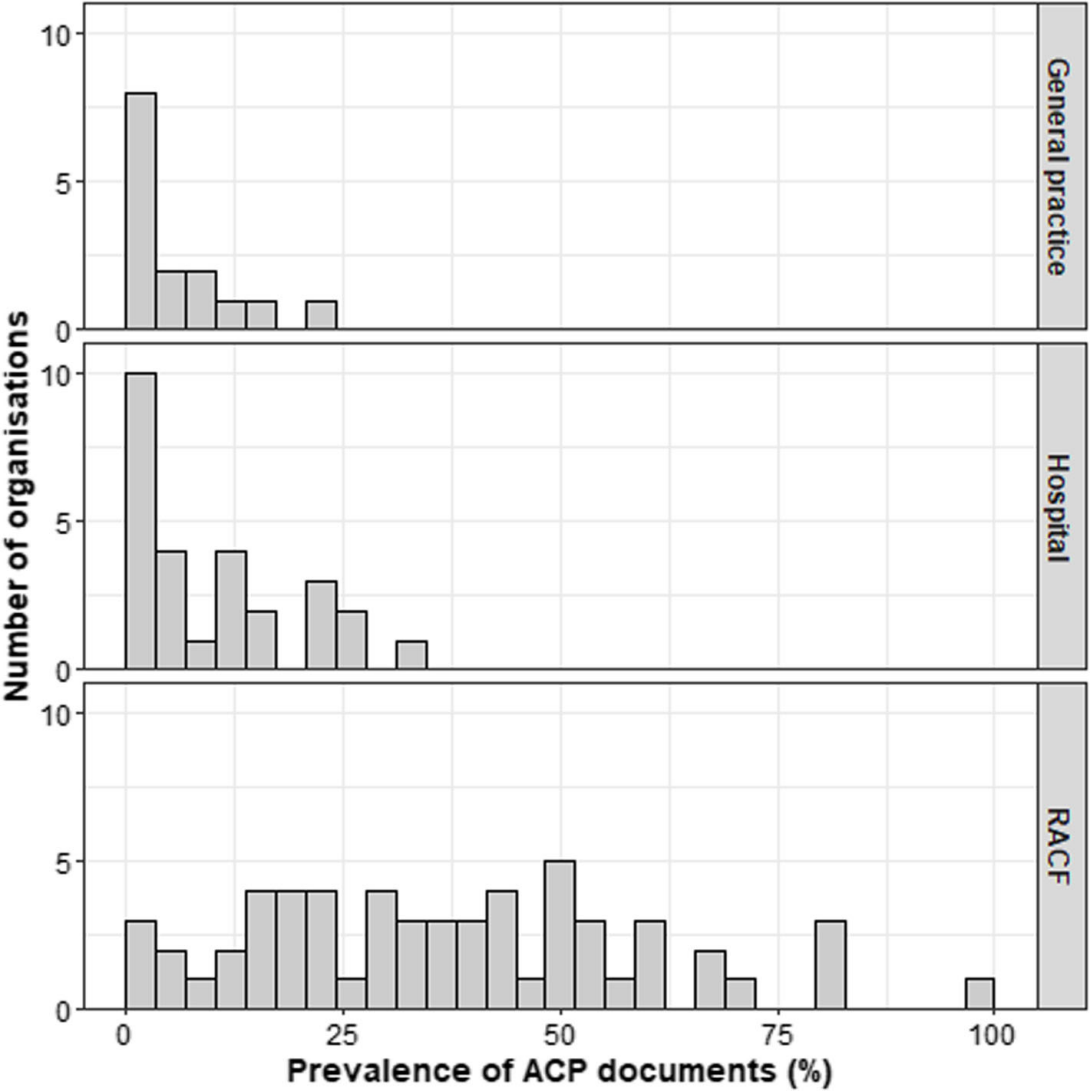
Histograms depicting frequency of participating organisations with different prevalence levels of advance care planning documents by sector. Note: bin width is approximately 3 percentage points, left most column reflects sites with 0–3% prevalence

Ordinary least-squares linear regression models were used to model ACD prevalence. As the organisation-specific prevalence variable was skewed (see Fig. 1), a log transformation was performed to satisfy the assumptions of this model class. A first stage of modelling derived unadjusted and adjusted prevalence estimates based on organisation characteristics (sector, jurisdiction, location, funding source) and a single variable capturing the presence of an ACP program (no program, implemented within three years, implemented more than 3 years ago). Means are estimated from the unadjusted models and back-transformed to the original prevalence scale and can be interpreted as such. Adjusted model means have also been provided, together with 95% confidence intervals (CI). The statistical significance level was set at *p* = 0.05. The adjusted model predicted prevalence rates significantly better than chance (*F*(15, 84) = 10.87, *p* < 0.001) with a multiple *R*^2^ = 0.66 and adjusted *R*^2^ = 0.60.

Separate follow up analyses were conducted for two sectors (hospital, RACF) to explore the influence of ACP program characteristics on ACD prevalence. General practice sites were not included in follow up analyses due to insufficient observations and low rates of ACP program implementation. Linear models were constructed for each sector in the same way as for the overall model. As with the overall model, log transformation of the outcome and initial characteristic selection to reduce the number of characteristics in the final models was performed, due to the smaller number of total sites for each type. For RACF sites, the adjusted model predicted prevalence rates significantly better than chance (*F*(11, 46) = 3.66, *p* < 0.001) with a multiple *R*^2^ = 0.47 and adjusted *R*^2^ = 0.34. The adjusted model for hospital sites was also significant (*F*(8, 18) = 5.33, *p* = 0.002), with multiple *R*^2^ = 0.70 and adjusted *R*^2^ = 0.57. Crude and adjusted model estimates are displayed for RACF and hospital sectors in Table 4.

## Results

### Organisations

Data collection occurred between June 2018 and January 2019. One hundred organisations participated in this study, of which 15 were general practices, 27 were hospitals, and 58 were RACFs. A total of 4187 patients/residents, with a median age of 82 years (Interquartile range = 14), had their health records audited.

Whilst all Australian jurisdictions were represented, only four (New South Wales, Queensland, Victoria and Western Australia) had organisations from each of the three sectors (general practice, hospital, RACF) represented. Organisations from metropolitan (*n* = 47, 47%), regional (*n* = 39, 39%) and rural/remote (*n* = 14, 14%) locations were included, and reported funding source included Government (*n* = 31, 31%), not for profit (*n* = 44, 44%) and private sources (*n* = 25, 25%). Organisations varied in size with general practices having a median full-time equivalent of five doctors and two nurses, and hospitals and RACFs having a median of 800 and 120 beds, respectively. Only 32% (*n* = 32) of organisations reported being able to access “My Health Record” [45], (highest in general practice (93%), followed by hospitals (59%) and very low in RACF (4%)) the remainder stating they could not (*n* = 40, 40%) or were unsure (*n* = 28, 28%). (Table 1).

**Table 1:** Organisation-level characteristics (*n* = 100)

Characteristic		GP	Hospital	RACF^#^	Overall	Location		
Total *n* (%)		15 (15)	27 (27)	58 (58)	100 (100)	Metropolitan	Regional	Rural/Remote
Jurisdiction	Australian Capital Territory	0 (0)	2 (7)	1 (2)	3 (3)	2 (67)	1 (33)	0 (0)
	New South Wales	1 (7)	8 (30)	20 (34)	29 (29)	14 (48)	14 (48)	1 (4)
	Northern Territory	5 (33)	1 (4)	0 (0)	6 (6)	1 (17)	0 (0)	5 (83)
	Queensland	1 (7)	7 (26)	14 (24)	22 (22)	10 (45)	11 (50)	1 (5)
	South Australia	2 (13)	0 (0)	7 (12)	9 (9)	2 (22)	4 (44)	3 (34)
	Tasmania	1 (7)	0 (0)	0 (0)	1 (1)	0 (0)	0 (0)	1 (100)
	Victoria	4 (26)	7 (26)	15(26)	26 (26)	16 (62)	8 (31)	2 (7)
	Western Australia	1 (7)	2 (7)	1 (2)	4 (4)	2 (50)	1 (25)	1 (25)
Location	Metropolitan	4 (27)	14 (52)	29 (50)	47 (47)			
	Regional	3 (20)	13 (48)	23 (40)	39 (39)			
	Rural/Remote	8 (53)	0 (0)	6 (10)	14 (14)			
Service Funding	Government	0 (0)	23 (85)	8 (14)	31 (31)			
	Not for profit	1 (7)	1 (4)	42 (72)	44 (44)			
	Private	14 (93)	3 (11)	8 (14)	25 (25)			
Size (Median, IQR^a^)	Doctor Full time equivalent	5, IQR =4.9	X	X	X			
	Nurse Full time equivalent	2, IQR 1.95	X	X	X			
	Number of beds	X	800, IQR = 270	120, IQR = 47.75	X			
Access to *My Health Record*^b^	Yes	14 (93)	16 (59)	2 (4)	32 (32)			
	No	0 (0)	8 (30)	32 (55)	40 (40)			
	Unsure	1 (7)	3 (11)	24 (41)	28 (28)			

*GP* General Practice, *RACF* Residential aged care facility

^a^ IQR Interquartile range

^b^Australian e-Health Record

### Advance care directive prevalence rate across organisations

The median ACD prevalence rate across all organisations was 19.4%, but prevalence varied considerably ranging from 0% (11 sites) to 100% (1 site). Figure 1 shows the variation in prevalence by organisation type, with the highest prevalence and widest variation present among RACFs, followed by hospitals and general practice. The mean prevalence (standard deviation) was 37.2% (17.3) for RACF, 10.8% (9.8) for hospitals and 5.6% (7.2) for general practice. The highest prevalence for each sector was 100% in RACF, 23% in general practice and 34% in hospitals.

### Advance care planning program-level characteristics

Most organisations reported having an ACP program (*n* = 81, 81%), training for staff (*n* = 76, 76%), written ACP information resources for patients/residents *(n* = 77, 77%), mechanisms to record the presence of an ACD (*n* = 100, 100%), and an ACP/ACD policy (*n* = 62, 62%). However, only 25 (25%) reported funding for ACP activities at their organisation. (Table 2).

**Table 2:** Advance care planning program-level characteristics

Characteristic		GP ***n*** (%)	Hospital ***n*** (%)	RACF ***n*** (%)	Overall ***n*** (%)
Total organisations		15 (15)	27 (27)	58 (58)	100 (100)
Does your organisation have an ACP program	Yes	10 (67)	19 (70)	52 (90)	81 (81)
	No	3 (20)	8 (30)	5 (8)	16 (16)
	Not sure	2 (13)	0 (0)	1 (2)	3 (3)
If yes, when program implemented^a^ (*n* = 81)	3 or more years	2 (20)	11 (58)	11(67)	48 (60)
	Within 3 years^a^	7 (70)	7 (37)	13 (25)	27 (33)
	Not sure	1 (10)	1 (5)	4 (8)	6 (7)
ACP training for staff available	Yes	9 (60)	24 (89)	43 (74)	76 (76)
	No	4 (27)	3 (11)	7 (12)	14 (14)
	Not sure	2 (13)	0 (0)	8 (14)	10 (10)
Presence of ACP /ACD policy	Yes	1 (7)	21(78)	40 (69)	62 (62)
	No	13 (86)	4 (15)	14 (24)	31 (31)
	Not sure	1 (7)	2 (7)	4 (7)	7 (7)
Written ACP resources for clients	Yes	7 (47)	24 (89)	46 (80)	77 (77)
	No	5 (33)	2 (7)	10 (17)	17 (17)
	Not sure	3 (20)	1 (4)	2 (3)	6 (6)
Mechanisms to record presence of an ACD	Yes	14 (93)	26 (97)	58 (100)	98 (98)
	No	1 (7)	0 (0)	0 (0)	1 (1)
	Not sure	0 (0)	1 (3)	0 (0)	1 (1)
Template ACD	Yes	4 (27)	22 (81)	45 (78)	71 (71)
	No	9 (60)	2 (7)	11 (19)	22 (22)
	Not sure	2 (13)	3 (12)	2 (3)	7 (7)
Funding available for ACP activities	Yes	0 (0)	13 (48)	12 (21)	25 (25)
	No	15 (100)	14 (52)	46 (79)	75 (75)
	Not sure	0 (0)	0 (0)	0 (0)	0 (0)
If Yes (*n* = 25), how is funding used? (one or more responses)					
Clinician facilitation of ACP conversations		X	13 (100)	4 (33)	17 (68)
Administration support for ACP		X	7 (54)	1 (8)	8 (32)
Recruit people /schedule ACP consultations		X	3 (23)	0 (0)	3 (12)
Clinical leadership/supervision		X	4 (31)	3 (25)	7 (28)
ACP education/ training		X	9 (69)	9 (75)	18 (72)

*ACP* advance care planning, *ACD* Advance Care Directive, *GP* general practice, *RACF* residential aged care facility

^a^ from date of data collection.

Among organisations reporting ACP programs, 33% (27/81) of these had been implemented within the three years (2015–2017) preceding data collection. Proportionately, hospitals (48%) were more likely than RACFs (21%) and general practices (0%) to have funding for ACP activities. Where funding was available to support ACP, this was most commonly directed towards ACP education and training (*n* = 18, 72%) and funding clinicians to facilitate ACP conversations (*n* = 17, 68%). However, the patterns of funding allocation differed across sector. Hospital sites with ACP funding more commonly allocated resources to clinicians to facilitate ACP conversations (*n* = 13, 100%), while the 12 RACF sites with ACP funding rarely allocated resources to ACP facilitation (*n* = 4, 33%) and more commonly provided resources for ACP education (*n* = 9, 75%).

### Organisational predictors of prevalence of advance care directives

A range of organisational characteristics were associated with ACD prevalence, in both unadjusted and adjusted models (see Table 3). Sector type was the most influential predictor, with RACF (unadjusted prevalence 28.7%, adjusted 20.6%) having much higher prevalence rates than either hospitals (unadjusted 6.4%, adjusted 5.8%) or general practices (unadjusted 2.5%, adjusted 6.6%). Regional sites (adjusted 11.0%) and rural and remote sites (adjusted 12.2%) had higher prevalence rates than metropolitan sites (adjusted 6.1%) in adjusted models only. Government funded (unadjusted 10.8%, adjusted 12.8%) and ‘not for profit’ (unadjusted 30.4%, adjusted 13.6%) organisations had higher prevalence rates than privately funded organisations (unadjusted 4.2%, adjusted 4.5%). Organisations with ACP programs implemented more than three years ago (unadjusted 23.6%, adjusted 14.4%) had higher prevalence rates than those with no program (unadjusted 5.7%, adjusted 6.3%) or more recently implemented programs (unadjusted 8.8%, adjusted 7.9%).

**Table 3:** Organisational predictors of prevalence of advance care directives

Characteristic	Level	Unadjusted results			Adjusted results		
		*Mean prevalence (%)	95% CI mean	***p***	^a^Mean prevalence (%)	95% CI mean	***p***
Type	General practice	2.5	(1.1, 4.8)	< 0.001	6.6	(3.1, 12.9)	< 0.001
	Hospital	6.4	(4.1, 9.8)		5.8	(3.1, 10.3)	
	RACF	28.7	(22.0, 37.4)		20.6	(13.1, 32.0)	
Jurisdiction	ACT	10.0	(1.9, 41.0)	0.001	9.9	(3.0, 28.9)	0.034
	New South Wales	13.2	(8.2, 20.9)		6.8	(4.2, 10.6)	
	Northern Territory	1.7	(0.0, 6.0)		4.3	(1.3, 11.4)	
	Queensland	24.1	(14.3, 40.3)		12.8	(7.7, 20.7)	
	South Australia	31.3	(13.9, 69.2)		16.5	(8.7, 30.7)	
	Tasmania	10.0	(0.1, 111.8)		22.5	(3.1, 132.2)	
	Victoria	14.1	(8.6, 22.8)		9.5	(5.8, 15.0)	
	Western Australia	2.0	(−0.1, 8.7)		4.0	(1.2, 13.5)	
Location	Metropolitan	11.9	(7.9, 17.7)	0.136	6.1	(3.7, 9.6)	0.012
	Regional	19.2	(12.5, 29.4)		11.0	(6.9, 17.2)	
	Rural or remote	9.3	(4.3, 18.8)		12.2	(6.7, 21.6)	
Service funding	Government	10.8	(7.1, 16.4)	< 0.001	12.8	(7.2, 22.2)	0.002
	Not for Profit	30.4	(21.8, 42.4)		13.6	(8.3, 22.0)	
	Private	4.2	(2.4, 7.0)		4.5	(2.6, 7.4)	
Year ACP program implemented	More than 3 years ago	23.6	(17.0, 32.7)	< 0.001	14.4	(9.4, 21.7)	0.01
	No program	5.7	(3.2, 9.7)		6.3	(3.7, 10.5)	
	Within 3 years	8.8	(4.9, 15.2)		7.9	(5.5, 14.1)	

^a^Prevalence values were calculated for individual sites based on the audit sample, but assuming they were representative of the full site before calculating mean prevalence. As this value is an estimate, 95% confidence intervals are included. This estimate was based on a log-transformation which was then back-transformed. *RACF* Residential aged care facility, *ACP* advance care planning, *ACT* Australian Capital Territory.

### Within sector type: predictors of prevalence of advance care directives

Given the large effect of sector type, separate analyses were conducted for hospital and RACF sectors (Table 4).

**Table 4:** Predictors of advance care directives in hospital and aged care facility organisations

Characteristic		Level	Unadjusted results			Adjusted results		
			^a^Mean prevalence %	95% CI mean	***p***	^a^Mean prevalence %	95% CI mean	***p***
**Residential aged care facilities**	State	ACT	17.8	(2.5, 99.1)	< 0.001	13.6	(2.1, 67.3)	0.22
		New South Wales	28.2	(19.1, 41.4)		22.1	(13.5, 35.9)	
		Queensland	30.2	(19.0, 47.8)		25.7	(15.0, 43.4)	
		South Australia	46.2	(24.1, 87.8)		22.1	(11.3, 42.3)	
		Victoria	26.4	(16.8, 41.2)		26.6	(16.3, 43.2)	
		Western Australia	3.7	(−0.1, 24)		2.6	(−0.2, 16.2)	
	Location	Metropolitan	20.3	(14.9, 27.6)	0.01	9.2	(5.2, 15.8)	0.008
		Regional	38.7	(27.5, 54.4)		18.0	(10.7, 29.7)	
		Rural or remote	48.0	(24.6, 93.0)		22.3	(10.3, 48.7)	
	Service funding	Government	32.0	(17.5, 57.9)	0.03	19.3	(9.5, 38.5)	< 0.001
		NFP	32.5	(25.0, 42.0)		26.5	(17.2, 40.8)	
		Private	13.4	(7.1, 24.7)		7.2	(3.3, 14.6)	
	Year ACP Program Implemented	More than three years ago	33.2	(25.2, 43.8)	0.13	18.2	(10.4, 31.6)	0.16
		No program	25.8	(14.3, 45.8)		20.1	(10.3, 38.4)	
		Within three years	17.9	(10.1, 31.0)		18.6	(4.9, 20.4)	
	Level of ACP Implementation	Low-medium (4 or less)	29.7	(21.3, 41.3)	0.77			
		High (5 or more)	27.8	(19.9, 38.6)				
**Hospitals**	^#^Jurisdiction	ACT	7.4	(1.9, 22.9)	< 0.001	5.3	(0.8, 20.9)	0.005
		New South Wales	1.2	(0.3, 2.7)		1.4	(0.2, 3.8)	
		Northern Territory	24.0	(4.7, 109.1)		35.3	(5.9, 188.3)	
		Queensland	17.9	(9.8, 32.0)		14.0	(6.7, 28.4)	
		Victoria	10.2	(5.4, 18.6)		8.8	(3.7, 19.4)	
		Western Australia	3.2	(0.5, 11.1)		3.4	(0.4, 12.8)	
	Location	Metropolitan	5.3	(2.3, 10.9)	0.46			
		Regional, Rural or remote	7.7	(3.7, 15.1)				
	Service Funding	Government	7.3	(4.1, 12.4)	0.49			
		Not for profit	3.2	(−0.6, 41.4)				
		Private	2.9	(0.0, 13.8)				
	My Health Record Access	No or unsure	5.6	(2.3, 12.4)	0.67			
		Yes	7.0	(3.5, 13.3)				
	Year ACP Program Implemented	More than three years ago	9.9	(5.4, 17.5)	0.02	8.2	(4.1, 15.7)	0.85
		No program	2.2	(0.7, 5.3)		6.1	(2.6, 13.0)	
		Within three years	11.7	(3.7, 33.5)		8.3	(2.3, 24.7)	
	Level of ACP Implementation	Low-medium (4 or less)	3.4	(1.1, 8.2)	0.09	6.0	(2.5, 12.8)	0.29
		High (5 or more)	8.6	(4.7, 15.1)		9.3	(5.1, 16.2)	

^a^Prevalence values were calculated for individual sites based on the sample of audited records, but assuming they were representative of the full site before calculating mean prevalence. As this value is an estimate, 95% confidence intervals are included. Model mean prevalence estimates are based on log-transformed prevalence values, which are then back-transformed. *ACP* Advance care planning, *ACT* Australian Capital Territory

Whilst several factors were significant in the unadjusted models, the predictors emerging as significant in adjusted models were different for hospital and RACF sectors. Within RACF sites, metropolitan sites (adjusted prevalence 9.2%) had lower prevalence rates than regional (adjusted 18.0%) or rural or remote (22.3%) sites. Privately funded RACF sites (adjusted prevalence 7.2%) had lower prevalence rates than Government (adjusted 19.3%) or Not For Profit (adjusted 26.5%) sites.

In hospitals, jurisdiction was associated with a higher ACD prevalence, with the Northern Territory (adjusted prevalence 35.3%) having the highest, and New South Wales (adjusted prevalence 1.4%) having the lowest. However, these results need to be interpreted with caution given jurisdictions did not have similar representation with no hospitals from Tasmania or South Australia, and only one hospital from the Northern Territory.

## Discussion

This study provides new evidence regarding organisational and ACP program characteristics associated with the prevalence of one or more ACDs at the point-of-care for older Australians in health and residential aged care services. Whilst the median ACD prevalence across all organisations was low (19%), rates varied greatly from 0 to 100%. Several organisational characteristics were associated with increased ACD prevalence rates on their own. However, when adjusted mean prevalence rates were considered, sector type was the strongest predictor, with RACFs having the highest prevalence (21%) compared with general practice (7%) and hospitals (6%). Most organisations reported having an ACP program, ACP training for staff, ACP resources and ACD templates for patients/residents, and mechanisms to record the presence of ACD(s). Only 25% of the study organisations reported having dedicated ACP funding available (none in GP organisations). Metropolitan sites, and privately funded sites, showed lower adjusted prevalence rates in the overall sample. In terms of ACP program implementation, while some aspects (e.g. time since implementation and overall level of implementation) were associated in unadjusted models, only the time since implementation was influential in the adjusted model for the overall sample.

Previous attempts to estimate ACD prevalence internationally have generally relied on self-report, been limited to one-type of ACD or have occurred in one region, healthcare setting, or the community [18, 25, 32–34, 46]. Similar to this study, prevalence rates vary across and within sectors. For example, previous ACD prevalence within aged care settings in Australia range from < 1 to 48% [23, 36]. Internationally, prevalence studies report rates of up to 59% in the USA, [47] and 44% in Canada, [48] 36% in Germany, [32] and 16% in Taiwan [49]. Similarly, hospital prevalence rates within Australia and internationally vary from < 1 to 41% [18, 23, 50–52]. Whilst studies on ACD prevalence rates in general practice are rare, rates of up 3–16% have been reported within Australian studies and 33% internationally [23, 53]. Thus, it might be expected that organisations included in this study have a range of ACD prevalence rates.

There is increasing evidence, including systematic reviews, that consider factors thought to be important for successful ACP implementation [13, 39, 41] within particular settings such as aged care [22, 36, 42] or primary care, [35] and in older populations [24, 40]. Whilst there is overlap and interaction between factors relevant to individual clients/patients, and providers and those that are more focused on the broader health system, in this study we aimed to specifically look at elements at the organisational and ACP program levels.

Consistent with existing literature, and considering the unadjusted results, services with an ACP program implemented over a longer timeframe (whether specifically funded or not), and higher numbers of ACP program components (e.g. ACP/ACD policy, staff training and a standardised ACD template) were associated with a higher organisation-specific ACD prevalence [13, 29, 35, 36, 38, 41, 42, 54]. Having funding available typically facilitates ACP uptake, [35, 38, 41, 54] and demonstrates an organisational commitment to ACP implementation. In the current study, when funding was available, it was commonly used for clinician facilitation of ACP discussions and ACP education and training for staff. Both of these activities are thought to be important factors for successful ACP implementation [18, 35, 36, 43, 54]. Funding was less frequently used for scheduling ACP consultations and clinical leadership. Leadership within an organisation can assist with setting the culture and expectations of staff and the organisation regarding ACP and can facilitate successful implementation into a service [13, 29, 35, 40, 43]. Importantly, in this study, all except one organisation reported a mechanism to alert the presence of an ACD. Lack of storage/retrieval mechanisms for ACDs are commonly reported as a barrier to successful ACP implementation across settings [13, 29, 35, 36, 38, 41, 42].

In this study, unadjusted results for jurisdiction showed a higher prevalence in South Australia and Queensland. In contrast, in adjusted results for the overall sample Tasmania showed the highest rates, although with large confidence intervals. For hospital sites in adjusted models Northern Territory had the highest rates. The results in the Northern Territory are supported by a recent publication outlining a detailed person-centred hospital program of ACP and goals of care [55]. However, given the lack of equal representation of all three sectors and jurisdictions, these results require further investigation. Within aged care settings, regional and rural organisations had a much higher prevalence than those in metropolitan areas. Rurality is positively associated with ACP discussions [56] and may be linked to important drivers for ACP such as concerns regarding place of care and place of death, [57] however more research is required to understand this association better.

Sector-type was the main organisation-level predictor of higher prevalence, with higher rates in RACFs compared to hospitals, and general practice. This result is consistent with findings from Australian and American studies [19, 23]. The higher prevalence seen in aged care settings might be expected given that their clients are generally older, unwell and frail; factors known to be associated with higher rates of ACP uptake and ACD completion [19, 23, 51]. The difference may also be explained as people in RACFs, as opposed to those in hospitals or attending GPs, are in their usual place of residence. However, despite the higher prevalence in RACFs than in hospitals and general practice, the adjusted mean prevalence is still very low at only 21%, suggesting there is still much work to be done. Similarly, people attending hospitals are often older, with significant and/or multiple illnesses which increase ACP/ACD completion. However competing demands in hospitals, lack of protected time for ACP, and the focus on curing patients make ACP activities more challenging [29, 35, 51, 54, 58]. The business of acute care may partly explain the association in hospitals between specific ACP funding availability and the higher prevalence of ACDs. In general practice, research has shown that whilst patients and clinicians are open to ACP, there are often other priorities and a lack of a systematic approach to care for older community-dwelling people, thus limiting ACP/ACD uptake [35, 40, 59].

Reasons for variability in prevalence rates, within this study, within countries and between nations is unclear, likely to be multifactorial and potentially relate to a range of factors either not measured in this study, and/or those measured with limited detail here. For example, in this study, we did not explore which staff provided ACP for their patients/residents, or how and when ACDs are completed. Likewise, we did not assess the type and length of ACP education or inquire about who was expected to participate, nor did we collect information regarding the role of leadership where this existed, and did not assess governance processes. Elsewhere we have examined the ACP policies and guidelines submitted by the organisations and found only 18 documents which are of high quality and currently in use across the sites [60]. The case-mix and acuity of the patients/residents attending these sites may have influenced clinical responses or workforce allocation at an organisational level. These are important factors that are likely to influence prevalence. Furthermore, qualitative interviews with organisations with very low and very high prevalence rates would likely add further important insights.

Synthesising the ever-increasing literature related to ACP implementation is challenging. In their scoping review on end-of-life care, Threapleton et al. [38] propose a conceptual framework to help organise ideas and provide a practical resource to support implementation, a framework which could be adapted for ACP implementation. Key elements are classified by whether they are macro-level (national context, policy, health system, legislation), meso-level (organisation-level) or micro-level (person – patient/ family member/ clinician) factors. In our study whilst we only assessed organisational (meso-level) characteristics associated with ACD prevalence, we recommend that future research consider how other factors such as national policy and legislation, and staff and patient/resident knowledge and attitudes interact with each other.

### Strengths and limitations

This study used a prospective multi-site audit methodology, a structured and standardised approach to data collection, including applicant self-reported information regarding organisation-level data at the time of recruitment to facilitate data collection. Study strengths included the large sample size and inclusion of organisations from all Australian jurisdictions, and across general practice, hospitals and residential aged care. Organisations included were diverse in terms of location, size and type of funding. However, recruitment was via an expression of interest process, and not all jurisdictions had each of the three sectors (general practice, hospitals and aged care) represented. These factors are likely to influence the generalisability of the findings. This study only collected information regarding ACDs, which is only one outcome of ACP discussions. Thus the prevalence of other ACP activity is unknown. As the study only allowed data collectors to search records for 15 min, a timeframe thought to be clinically appropriate, some ACDs may have been missed. This study also did not measure whether completed ACDs influenced the care received. Small cell counts in the data from some organisations may have impacted on the model fit. The content and quality of ACP facilitation, education programs, patient/resident resources and ACD templates used by sites were not assessed.

## Conclusions

This study is the first attempt to quantify organisation-level and ACP program characteristics associated with ACD prevalence for older Australians across all jurisdictions and three sectors. The median ACD prevalence across all organisations was low at 19%, with substantial variation by site. Sector type was the strongest predictor, with RACFs having the highest prevalence compared with general practice and hospitals. The low prevalence rates overall and in all three settings have important implications for sector-wide system improvement, if the true value of ACP is to be realised. Conceptualising ACP as an iterative process over the person’s health journey, within and between settings and over multiple interactions with these services is essential. Ideally ACP should be commenced early in the community, and reviewed regularly as a person’s health and social situation changes, thus highlighting that all three sectors studied here have interconnected and critical roles to play in the process.

ACP implementation is complex and requires appropriate support from health and aged care organisations. As the first quantitative study of its type, it is hoped these findings (and the methodology adopted) provide a basis to support the future implementation of ACP. In addition, having examined organisation-level and ACP program characteristics, this work provides a basis for supporting further research.

## Data Availability

The datasets generated and/or analysed during the current study are not publicly available due to privacy reasons and ethical restrictions but are available from the corresponding author on reasonable request.

## References

[1] Global, regional, and national incidence, prevalence, and years lived with disability for 328 diseases and injuries for 195 countries, 1990–2016 (2017). A systematic analysis for the Global Burden of Disease Study 2016. Lancet.

[2] Swerissen H, Duckett S. Dying Well: Grattan Institute; 2014. ISBN: 978–1–925015-61-4

[3] Institute of Medicine (2014). Dying in America: Improving Quality and Honoring Individual Preferences Near the End of Life.

[4] Australian Government Department of Health and Ageing (2018). National Palliative Care Strategy 2018.

[5] Aged Care Quality and Safety Commission Guidance and Resources for Providers to support the Aged Care Quality Standards. 2019.

[6] Australian Commission on Safety and Quality in Healthcare: National Standards https://www.safetyandquality.gov.au/. 2020.

[7] The Clinical Technical and Ethical Principal Committee of the Australian Health Minister's Advisory Council (2011). A National Framework for Advance Care Directives.

[8] Royal Commission into Aged Care Quality and Safety, Commonwealth Of Australia (2019). Advance Care Planning in Australia. Background Paper 5. June 2019.

[9] Haining C, Nolte LDKM (2020). Australian advance care planning laws: can we improve consistency?.

[10] Sudore RL, Lum HD, You JJ, Hanson LC, Meier DE, Pantilat SZ, et al. Defining Advance Care Planning for Adults: A Consensus Definition From a Multidisciplinary Delphi Panel. J Pain Symptom Manage. 2017. 10.1016/j.jpainsymman.2016.12.331 PMC5728651 28062339

[11] Rietjens JAC, Sudore RL, Connolly M, van Delden JJ, Drickamer MA, Droger M (2017). Definition and recommendations for advance care planning: an international consensus supported by the European Association for Palliative Care. Lancet Oncol..

[12] Sudore RL, Heyland DK, Lum HD, Rietjens JAC, Korfage IJ, Ritchie CS (2018). Outcomes That Define Successful Advance Care Planning: A Delphi Panel Consensus. J Pain Symptom Manage..

[13] Jimenez G, Tan WS, Virk AK, Low CK, Car J, Ho AHY (2018). Overview of Systematic Reviews of Advance Care Planning: Summary of Evidence and Global Lessons. J Pain Symptom Manage..

[14] Brinkman-Stoppelenburg A, Rietjens JA, van der Heide A (2014). The effects of advance care planning on end-of-life care: A systematic review. Palliat Med..

[15] Houben CH, Spruit MA, Groenen MT, Wouters EF, Janssen DJ (2014). Efficacy of advance care planning: a systematic review and meta-analysis. J Am Med Directors Assoc.

[16] Hartog CS, Reinhart K (2018). Staff and family response to end-of-life care in the ICU. Curr Opin Anaesthesiol..

[17] Klinger CA, Howell D, Zakus D, Deber RB (2014). Barriers and facilitators to care for the terminally ill: A cross-country case comparison study of Canada, England, Germany, and the United States. Palliative Medicine..

[18] Detering KM, Hancock AD, Reade MC, Silvester W (2010). The impact of advance care planning on end of life care in elderly patients: randomised controlled trial. BMJ..

[19] Yadav KN, Gabler NB, Cooney E, Kent S, Kim J, Herbst N (2017). Approximately one in three US adults completes any type of advance directive for end-of-life care. Health Affairs..

[20] Buck K, Detering KM, Pollard A, Sellars M, Ruseckaite R, Kelly H, et al. Concordance between self-reported completion of advance care planning documentation and availability of documentation in Australian health and residential aged care services. J Pain Symptom Manage. 2019. 10.1016/j.jpainsymman.2019.04.026 31029805

[21] Hemsley B, Meredith J, Bryant L, Wilson NJ, Higgins I, Georgiou A, et al. An integrative review of stakeholder views on Advance Care Directives (ACD): Barriers and facilitators to initiation, documentation, storage, and implementation. Patient Educ Couns. 2019. 10.1016/j.pec.2019.01.007 30799141

[22] Flo E, Husebo BS, Bruusgaard P, Gjerberg E, Thoresen L, Lillemoen L (2016). A review of the implementation and research strategies of advance care planning in nursing homes. BMC Geriatr..

[23] Detering KM, Buck K, Ruseckaite R, Kelly H, Sellars M, Sinclair C (2019). Prevalence and correlates of advance care directives among older Australians accessing health and residential aged care services: multicentre audit study. BMJ Open..

[24] Sharp T, Moran E, Kuhn I, Barclay S. Do the elderly have a voice? Advance care planning discussions with frail and older individuals: a systematic literature review and narrative synthesis. Br J Gen Pract. 2013;63. 10.3399/bjgp13X673667 PMC3782798 24152480

[25] Yung VY, Walling AM, Min L, Wenger NS, Ganz DA (2010). Documentation of advance care planning for community-dwelling elders. Journal of palliative medicine..

[26] Thomas K, Lobo B, Detering K (2017). Advance care planning in end of life care.

[27] Aguilera V (2020). Conversations that go unspoken: The necessity of advance care planning. Nursing..

[28] Waller A, Sanson-Fisher R, Nair BR, Evans T (2020). Preferences for End-of-Life Care and Decision Making Among Older and Seriously Ill Inpatients: A Cross-Sectional Study. J Pain Symptom Manage..

[29] Hagen NA, Howlett J, Sharma NC, Biondo P, Holroyd-Leduc J, Fassbender K (2015). Advance care planning: identifying system-specific barriers and facilitators. Curr Oncol..

[30] Biondo PD, Lee LD, Davison SN, Simon JE (2016). Advance Care Planning Collaborative R, Innovation Opportunities P. How healthcare systems evaluate their advance care planning initiatives: Results from a systematic review. Palliat Med..

[31] Rhee JJ, Zwar NA, Kemp LA (2012). Uptake and implementation of Advance Care Planning in Australia: findings of key informant interviews. Australian Health Review..

[32] in der Schmitten J, Lex K, Mellert C, Rothärmel S, Wegscheider K, Marckmann G (2014). Implementing an advance care planning program in German nursing homes: results of an inter-regionally controlled intervention trial. Dtsch Ärztebl Int.

[33] Hammes BJ, Rooney BL, Gundrum JD (2010). A comparative, retrospective, observational study of the prevalence, availability, and specificity of advance care plans in a county that implemented an advance care planning microsystem. J Am Geriatr Soc.

[34] Heyland DK, Barwich D, Pichora D, Dodek P, Lamontagne F, You JJ (2013). Failure to engage hospitalized elderly patients and their families in advance care planning. J Am Med Assoc Intern Med.

[35] Risk J, Mohammadi L, Rhee J, Walters L, Ward PR (2019). Barriers, enablers and initiatives for uptake of advance care planning in general practice: a systematic review and critical interpretive synthesis. BMJ Open..

[36] Batchelor F, Hwang K, Haralambous B, Fearn M, Mackell P, Nolte L (2019). Facilitators and barriers to advance care planning implementation in Australian aged care settings: A systematic review and thematic analysis. Australas J Ageing..

[37] Lovell A, Yates P. Advance Care Planning in palliative care: A systematic literature review of the contextual factors influencing its uptake 2008–2012. Palliat Med. 2014. 10.1177/0269216314531313 24821708

[38] Threapleton DE, Chung RY, Wong SYS, Wong ELY, Kiang N, Chau PYK (2017). Care Toward the End of Life in Older Populations and Its Implementation Facilitators and Barriers: A Scoping Review. J Am Med Directors Assoc.

[39] Jimenez G, Tan WS, Virk AK, Low CK, Car J, Ho AHY. State of advance care planning research: A descriptive overview of systematic reviews. Palliat Support Care. 2018:1–11. 10.1017/S1478951518000500 30058506

[40] Combes S, Nicholson CJ, Gillett K, Norton C (2019). Implementing advance care planning with community-dwelling frail elders requires a system-wide approach: An integrative review applying a behaviour change model. Palliat Med..

[41] Hemsley B, Meredith J, Bryant L, Wilson NJ, Higgins I, Georgiou A (2019). An integrative review of stakeholder views on Advance Care Directives (ACD): Barriers and facilitators to initiation, documentation, storage, and implementation. Patient Educ Couns..

[42] Gilissen J, Pivodic L, Smets T, Gastmans C, Vander Stichele R, Deliens L (2017). Preconditions for successful advance care planning in nursing homes: A systematic review. Int J Nurs Stud.

[43] Chan CWH, Ng NHY, Chan HYL, Wong MMH, Chow KM (2019). A systematic review of the effects of advance care planning facilitators training programs. BMC Health Serv Res..

[44] Detering KM, Buck K, Sellars M, Kelly H, Sinclair C, White B (2019). Prospective multicentre cross-sectional audit among older Australians accessing health and residential aged care services: protocol for a national advance care directive prevalence study. BMJ Open..

[45] Australian Digital Health Agency. My Health Record. https://www.myhealthrecord.gov.au/.

[46] White B, Tilse C, Wilson J, Rosenman L, Strub T, Feeney R (2014). Prevalence and predictors of advance directives in Australia. Intern Med J..

[47] McAuley WJ, Buchanan RJ, Travis SS, Wang S, Kim M (2006). Recent trends in advance directives at nursing home admission and one year after admission. Gerontol.

[48] Siu HYH, Elston D, Arora N, Vahrmeyer A, Kaasalainen S, Chidwick P (2020). The Impact of Prior Advance Care Planning Documentation on End-of-Life Care Provision in Long-Term Care. Can Geriatr J..

[49] Lo Y-T, Wang J-J, Liu L-F, Wang C-N (2010). Prevalence and related factors of do-not-resuscitate directives among nursing home residents in Taiwan. J Am Med Directors Assoc.

[50] Scott IA, Rajakaruna N, Shah D, Miller L, Reymond E, Daly M (2016). Normalising advance care planning in a general medicine service of a tertiary hospital: an exploratory study. Aust Health Rev..

[51] Knight T, Malyon A, Fritz Z, Subbe C, Cooksley T, Holland M (2020). Advance care planning in patients referred to hospital for acute medical care: results of a national day of care survey. EClinicalMedicine..

[52] Barnato AE, O'Malley AJ, Skinner JS, Birkmeyer JD (2019). Use of Advance Care Planning Billing Codes for Hospitalized Older Adults at High Risk of Dying: A National Observational Study. J Hosp Med..

[53] Meeussen K, Van den Block L, Echteld M, Bossuyt N, Bilsen J, Van Casteren V (2011). Advance care planning in Belgium and The Netherlands: a nationwide retrospective study via sentinel networks of general practitioners. Journal of pain and symptom management..

[54] Dixon J, Knapp M (2018). Whose job? The staffing of advance care planning support in twelve international healthcare organizations: a qualitative interview study. Bmc Palliative Care.

[55] Spencer E, Waran E (2020). Opening the lines of communication: towards shared decision making and improved end-of-life care in the Top End. Med J Aust..

[56] Lam LAA, Baquir PJ, Chowdhury N, Tran K, Bailey J. Current practices, barriers and enablers for advance care planning among healthcare workers of aged care facilities in western New South Wales, Australia. Rural and Remote Health. 2018;18(4714). 10.22605/RRH4714 30447659

[57] Fletcher S, Sinclair C, Rhee J, Goh D, Auret K (2016). Rural health professionals' experiences in implementing advance care planning: a focus group study. Aust J Prim Health..

[58] Tan WS, Car J, Lall P, Low CK, Ho AHY (2019). Implementing Advance Care Planning in Acute Hospitals: Leading the Transformation of Norms. J Am Geriatr Soc..

[59] Glaudemans JJ (2015). Moll van Charante EP, Willems DL. Advance care planning in primary care, only for severely ill patients? A structured review. Fam Pract..

[60] Macleod A, Detering K, Nolte L (2020). Content and quality assessment of advance care planning policies in Australian health and residential aged care services:Implications for future policy development.

